# Infection Cycle of *Artichoke Italian Latent Virus* in Tobacco Plants: Meristem Invasion and Recovery from Disease Symptoms

**DOI:** 10.1371/journal.pone.0099446

**Published:** 2014-06-09

**Authors:** Elisa Santovito, Tiziana Mascia, Shahid A. Siddiqui, Serena Anna Minutillo, Jari P. T. Valkonen, Donato Gallitelli

**Affiliations:** 1 Dipartimento di Scienze del Suolo, della Pianta e degli Alimenti, Università degli Studi di Bari Aldo Moro, Bari, Italy; 2 Istituto di Virologia vegetale del Consiglio Nazionale della Ricerca, Unità Operativa di Supporto di Bari, Bari, Italy; 3 Department of Agricultural Sciences, University of Helsinki, Helsinki, Finland; The Ohio State University, United States of America

## Abstract

Nepoviral infections induce recovery in fully expanded leaves but persist in shoot apical meristem (SAM) by a largely unknown mechanism. The dynamics of infection of a grapevine isolate of *Artichoke Italian latent virus* (AILV-V, genus *Nepovirus*) in tobacco plants, including colonization of SAM, symptom induction and subsequent recovery of mature leaves from symptoms, were characterized. AILV-V moved from the inoculated leaves systemically and invaded SAM in 7 days post-inoculation (dpi), remaining detectable in SAM at least up to 40 dpi. The new top leaves recovered from viral symptoms earliest at 21 dpi. Accumulation of viral RNA to a threshold level was required to trigger the overexpression of *RDR6* and *DCL4*. Consequently, accumulation of viral RNA decreased in the systemically infected leaves, reaching the lowest concentration in the 3rd and 4th leaves at 23 dpi, which was concomitant with recovery of the younger, upper leaves from disease symptoms. No evidence of virus replication was found in the recovered leaves, but they contained infectious virus particles and were protected against re-inoculation with AILV-V. In this study we also showed that AILV-V did not suppress initiation or maintenance of RNA silencing in transgenic plants, but was able to interfere with the cell-to-cell movement of the RNA silencing signal. Our results suggest that AILV-V entrance in SAM and activation of RNA silencing may be distinct processes since the latter is triggered in fully expanded leaves by the accumulation of viral RNA above a threshold level rather than by virus entrance in SAM.

## Introduction

Procedures to obtain virus-free planting material can provide novel cellular and molecular insights in the study of virus-plant interactions [1], [2]. Shoot apical meristem (SAM) culture preceded or followed by heat therapy is widely used to produce virus-free plants although its efficiency in virus eradication depends on the virus and the host genotype [1], [3], [4], [5]. During the sanitation of the reflowering globe artichoke variety “Brindisino” with mixed infection of *Artichoke Italian latent virus* (AILV) genus *Nepovirus*, family *Secoviridae* [6] and *Artichoke latent virus* (ArLV), genus *Potyvirus*, family *Potyviridae* [7], ArLV was eliminated by means of SAM culture, while AILV was removed only when two rounds of SAM culture were spaced out with *in vitro* thermotherapy [2]. These preliminary results suggested that AILV was able to enter SAM at some stage of infection and to persist therein. This hypothesis is in line also with the previous finding that AILV is transmitted through seeds of globe artichoke [8], a process that would be dependent on the presence of virus in meristems [9], [10].

SAM is a strong photosynthetic sink thus being an ideal destination of viruses although most of them are excluded from there [11], [12]. It has been proposed that meristem exclusion is a variation of the RNA silencing (RS)-related “recovery” process that is restricted to the growing point of the infected plant and regulates selectively the entry and persistence of RNA in the shoot apex, including viruses and long-distance post-transcriptional gene silencing (PTGS) signals [13]. On the contrary, the “classical recovery” would be meristem exclusion that operates not only in the meristem but also in the uppermost leaves of the plant that remain free of symptoms [14], [15]. In line with this proposal, it was demonstrated that *Cucumber mosaic virus* (CMV) and *Tobacco rattle virus* (TRV) can transiently infect meristem tissues, in contrast to *Potato virus X* (PVX), and for persistence they would need to fully suppress RS implicated in the meristem exclusion process [13], [14], [16]. The exclusion of PVX would implicate a silencing mechanism that initiates in lower uninfected tissues, moves at or ahead the infection front and involves a long-range RS signaling regulated by host RNA-dependent RNA polymerase RDR6 [14]. Conversely, in plants with SAM transiently infected with TRV and CMV, the priming of RS would require the transient presence of these viruses in SAM and would be independent from the activity of *RDR*6 [16], [17], [18]. The model proposed by Martín-Hernández and Baulcombe [16] suggests also that a “transient accumulation mechanism” would operate in plants infected by TRV and would affect virus accumulation in leaves developed during or after the transient phase of meristem invasion. In the leaves derived from the transiently infected meristem, the virus would persist and continue to accumulate while the leaves derived from virus-free meristem from the post-transient phase exhibit very low virus levels.

In infected plants, recovery usually refers to the condition of new emerging leaves, which develop without symptoms, may contain low concentration of the virus and resist to further virus infection through a sequence-specific RS mechanism, while the infected symptomatic leaves remain virus-infected and continue to show disease symptoms. The first studies linking recovery of plants from viral infection with RS were carried out on nepoviruses [15], but only few of them have addressed viral distribution in SAM in the context of recovery. Dong *et al.* [19] studied dynamics of *Tobacco ringspot virus* (TRSV) distribution in the SAM and in the root apical meristem of tobacco, coming to the conclusion that TRSV persisted in the SAM of tobacco and the asymptomatic leaves of *Nicotiana benthamiana*, but it was transient in the root apical meristem and asymptomatic leaves of tobacco plants. Siddiqui *et al.* [10] reported that transgenic expression of some VSR affected the temperature-dependent infection pattern of the TRSV potato calico strain but no information was provided on the effect of such VSR on the recovery phenotype of TRSV-infected tobacco plants. Jovel *et al.* [20] characterized the interaction of *Tomato ringspot virus* (ToRSV) with host defense responses during symptom induction and subsequent recovery showing the activation of RS, which, however, did not reduce virus titer.

AILV is a member of subgroup B of the genus *Nepovirus* [6], has isometric particles c. 30 nm in diameter, sedimenting as three components with coefficient of 55S (T), 95S (M), and 121S (B). M and B particles, each encapsidate one species of functional single-stranded RNA with estimated Mr of 1.5×10^6^ (RNA-2) and 2.4×10^6^ (RNA-1), respectively [21], [22] but only a partial nucleotide sequence of RNA-2 is available (Acc. No. X87254). The aim of the present study was to better understand the molecular mechanisms behind invasion and persistence of a grapevine isolate of AILV (denoted AILV-V) in SAM, using infected tobacco plants as an amenable experimental system facilitating the study. Time-course experiments were carried out to estimate dynamics of viral RNA accumulation from inoculation up to two months after infection during different stages of tobacco plant growth. Data were related also with virus entrance, distribution and persistence in tobacco SAM, development of disease symptoms and ability of AILV-V to interfere with the RS-based defense response activated in infected plants.

## Results

### AILV-V Infection in *N. tabacum* Induces Severe Symptoms Followed by Complete Recovery, Undetectable Virus Replication and Resistance to Secondary Infection in Recovered Leaves

**Figure 1:**
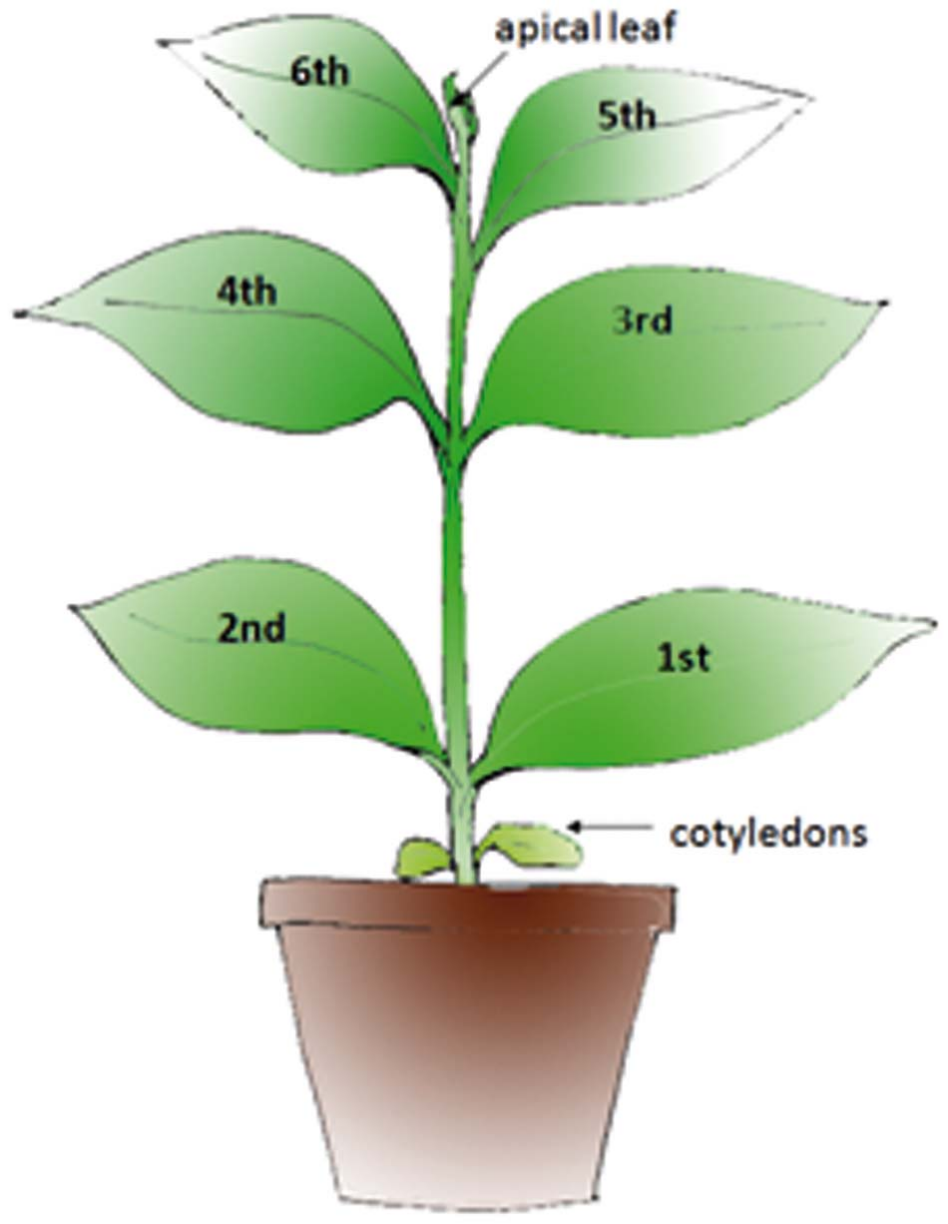
Scheme of a Samsun tobacco plant showing position of leaves used in this study.

**Figure 2:**
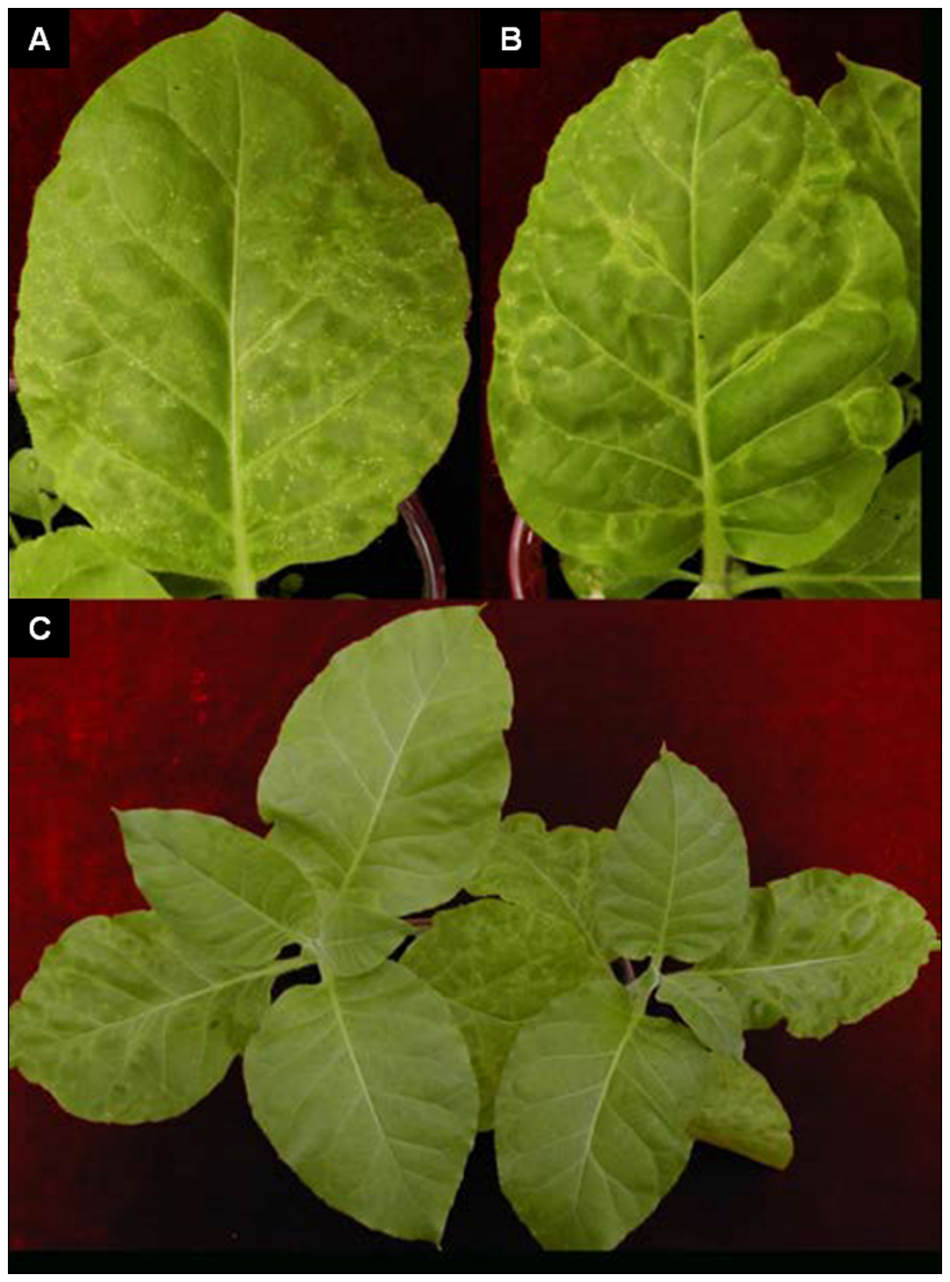
Samsun tobacco plants recover from disease symptoms induced by AILV-V. In **A,** local symptoms in inoculated leaves appeared by 7 dpi and consisted in chlorotic spots, small necrotic rings and line patterns etched in leaf epidermis. In **B,** systemic symptoms are shown in the 3rd and 4th leaf and consist in chlorotic or necrotic ringspots surrounding the veins and peripheral vein clearing. In **C,** young leaves emerged between 21 and 28 dpi showing the recovery phenotype from disease symptoms.

Local symptoms of AILV-V infection appeared within 7 days post inoculation (dpi) in inoculated leaves (i.e., in 1st and 2nd fully expanded true leaves; Figure 1) of tobacco plants and consisted of small necrotic rings and line patterns etched in leaf epidermis (Figure 2A), followed by the appearance of chlorotic spots that turned necrotic as leaf blade enlarged. Overall local symptoms were severe and caused pronounced leaf blade distortion and precocious senescence. Systemic symptoms appeared in the 3rd and 4th true leaves and reached maximum severity at 14 dpi. They consisted in chlorotic/necrotic ringspots surrounding the veins, peripheral vein clearing and necrotic pinpoints and severe deformation of leaf blade and margin (Figure 2B). After this severe symptom expression, plants initiated a rapid recovery and by 28 dpi the 5th and 6th unfolded true leaves were free of symptoms (Figure 2C) and plants grew normally up to 60 dpi.

**Figure 3:**
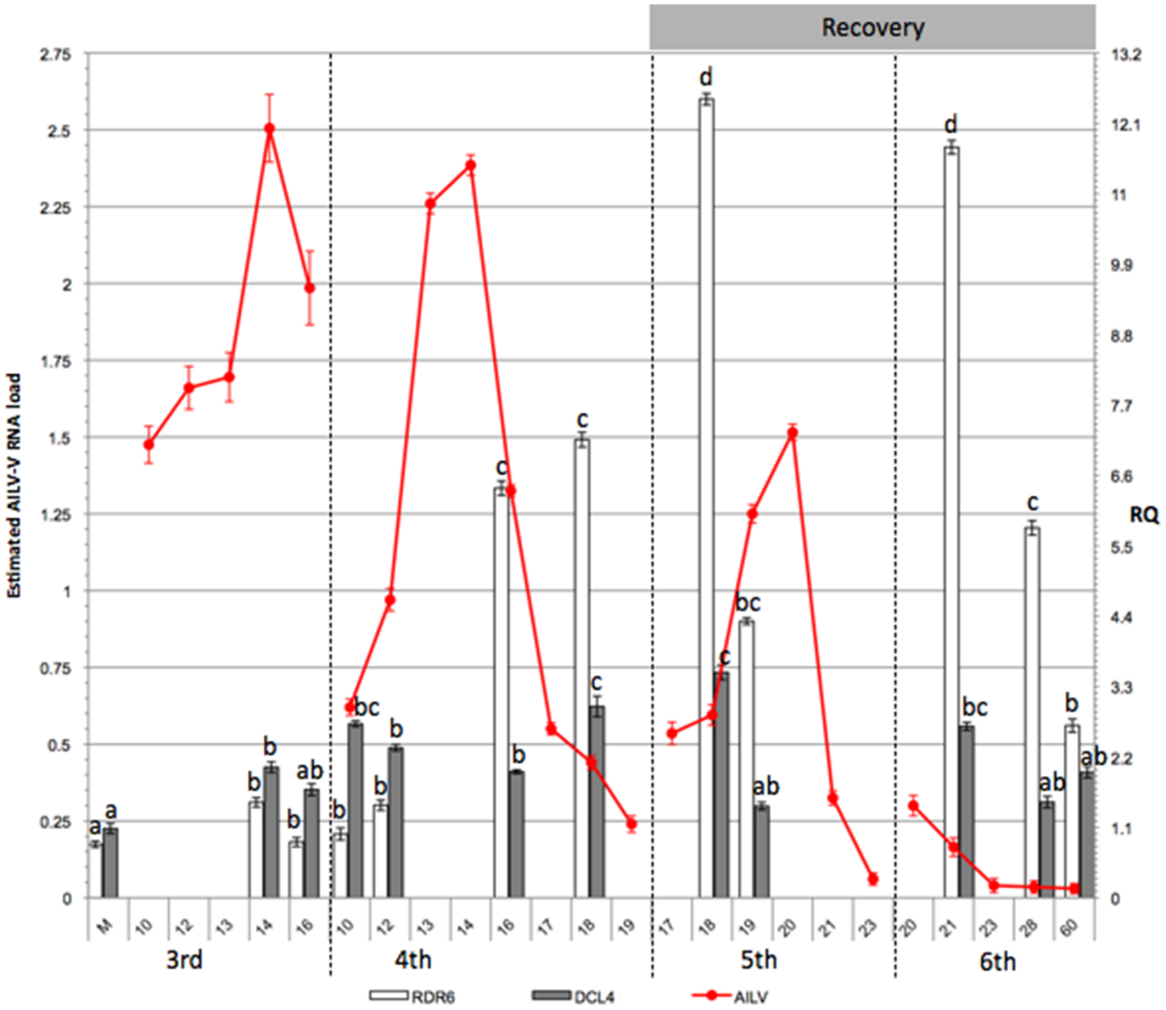
AILV-V RNA-2 accumulation varies in the same leaf, progressively decreasing moving to the successive leaf. Load of viral RNA (lines) was estimated by quantitative dot blot hybridization. RNA data are expressed as means of two independent experiments, were derived from spot intensity values of the target RNAs and were calculated on the basis of a standard curve generated by serial dilutions of a plasmid preparation containing the fragment of the RNA-2 of AILV-V targeted by the probe. Samples were collected from the 3^rd^, 4^th^, 5^th^, and 6^th^ leaves at 24 h intervals from 10 to 23 dpi and then at 28 and 60 dpi. Each point in the line chart represents average of three biological replicates for each of the two experiments and error bars on lines represent the standard error among replicates. Figure shows also the relative quantity (RQ) of *RDR6* and *DCL4* transcripts (columns chart) in samples of tobacco plants collected at selected time points between 10 and 60 dpi with AILV-V. The values were first normalized on the accumulation level of the *GAPDH* mRNA (Δ cycle threshold [Ct] = Ct_GAPDH_–Ct_target_ RNA) and then used to determine the relative quantification of each target RNA with a calibrator, according to the formula ΔΔCt = ΔCt_calibrator_–ΔCt_target_ RNA. Each target mRNA in an individual mock-inoculated plant served as calibrator (RQ set to 1) for the respective gene. RQ for *RDR6* and *DCL4* transcripts was deduced by the formula expression 2^−ΔΔCt^. Columns represent mean RQ values from three biological replicates for each of the two experiments and different letters represent statistically significant differences values according to separate one-way ANOVA analysis for each target mRNA, using Tukey's test (P<0 05). Vertical bars on columns represent standard deviations among replicates.

**Figure 4:**
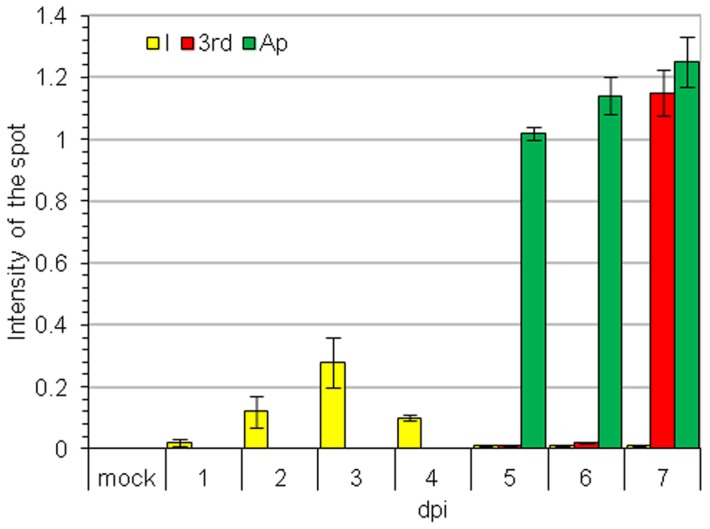
From the inoculated leaf, AILV-V moves first into shoot topmost leaf and then to lower leaves. Dot blot hybridization of samples collected from leaves of tobacco plants from 1 to 7-V. Plants at the 1103 growth stage according to the scale for coding growth stages in tobacco – coresta (i.e. with the 3rd leaf unfolded) were inoculated mechanically on the 1st and 2nd leaf. Columns represent mean values of the intensity of dot blot hybridization signal from two technical replicates of samples collected from two plants at each time point. The intensity of hybridization signal with antisense probe to detect viral genome (+)RNA was estimated from serial dilutions of a plasmid preparation, containing the fragment of the RNA-2 of AILV-V targeted by the probe. Vertical bars represent the standard error. I =  inoculated leaf; A =  apical leaf of the shoot tip. 3rd =  third leaf i.e. the first leaf above the two basal leaves used for rub inoculation; Mock  =  leaf mock-inoculated with buffer.

Accumulation of AILV-V RNA was estimated in two experiments (mean values shown in Figure 3 and Table 1), each carried out on 18 plants. Leaf disks (50 mg) were collected from 3rd to 6th true leaves of three plants, representing three biological replicates for each time point. Samples collected between 10 and 23 dpi showed a cyclic variation of AILV-V RNA loads in each leaf, reaching the highest accumulation at 14 dpi in the 3rd leaf (Figure 3 and Table 1) when the maximum symptom severity was observed. Viral RNA concentration was progressively less in the 4th, 5th and 6th leaf to reach a steady-state condition that was maintained up to 60 dpi (Figure 3). Accumulation of AILV-V-RNA was also analyzed daily from 1 to 7 dpi in the samples collected from the inoculated 1st and 2nd unfolded true leaves, the topmost leaf (apical leaf in Figure 1) and the 3rd true leaf (the 3rd leaf in Figure 1). Samples were collected from four plants. To avoid biases in selecting the portion of leaf tissues to be analyzed, due to difference in size between apical and mature leaves, the whole leaf was harvested and crushed with a roller press in six vol (v/w) of alkaline solution and aliquots (5 µl) were used for quantitative dot blot hybridization. Figure 4 shows that viral RNA was detectable in inoculated leaves by 2 dpi and in the apical leaf by 5 dpi, while it was found in the 3rd leaf not earlier than 7 dpi. This pattern of the systemic movement of AILV-V is consistent with phloem transport of photoassimilates from source to sink organs, as shown for TMV in *N. tabacum* [23] and *N. benthamiana* [24].

**Table 1:** Comparison of accumulation of viral RNAs and expression level of *RDR6* and *DCL4* in time course experiments with tobacco plants challenged with AILV-V, PVY-SON41 or both viruses.

	Estimated viral RNA Load†					RQ†					
Leaf position	Sampling time (dpi)	AILV-V*	PVY-SON41**	AILV-V+PVY-SON41		AILV-V *		PVY-SON41 **		AILV-V+PVY-SON41	
				AILV-V	PVY-SON41	*RDR6*	*DCL4*	*RDR6*	*DCL4*	*RDR6*	*DCL4*
**mock**	10	0.00±0.00	0.00±0.00	0.00±0.00	0.00±0.00	0.84±0.05 (a)	1.08±0.08 (a)	1.01±0.14 (a)	0.99±0.21 (a)	1.00±0.06 (a)	1.01±0.08 (a)
**3rd**	14	2.51±0.05	16.62±0.72	0.13±0.03	3.04±0.08	1.49±0.07 (b)	2.04±0.08 (b)	2.37±0.24 (a)	2.03±0.20 (ab)	1.80±0.07 (a)	0.31±0.06 (a)
	16	1.99±0.05	18.94±0.57	0.16±0.05	4.62±0.08	0.87±0.07 (b)	1.69±0.09 (ab)	3.46±0.29 (a)	2.50±0.18 (ab)	1.33±0.09 (a)	0.58±0.04 (a)
**4th**	10	0.62±0.03	3.88±0.32	0.20±0.02	0.60±0.09	1.00±0.09 (b)	2.72±0.05 (bc)	20.98±0.33 (bc)	3.49±0.38 (bc)	1.71±0.07 (a)	2.42±0.07 (c)
	12	0.97±0.04	9.85±0.75	0.26±0.03	1.25±0.09	1.45±0.08(b)	1.97±0.05 (b)	34.58±0.44 (d)	3.04±0.28 (bc)	9.57±0.06 (c)	8.18±0.10 (e)
	16	1.33±0.02	36.57±0.78	0.61±0.04	9.07±0.31	6.40±0.11 (c)	1.99±0.03 (b)	29.76±0.29 (cd)	5.20±0.21 (d)	14.94±0.09 (d)	4.30±0.17 (d)
	18	0.44±0.02	46.57±0.93	1.30±0.04	11.59±0.31	7.16±0.12 (c)	2.99±0.16 (c)	67.82±0.47 (e)	4.25±0.22(cd)	1.00±0.08 (a)	1.61±0.12 (bc)
**5th**	18	0.60±0.03	21.13±0.99	1.09±0.05	12.10±0.35	12.48±0.09 (d)	3.52±0.12 (c)	25.82±0.50 (bcd)	4.15±0.32 (cd)	5.07±0.16 (b)	4.30±0.10 (d)
	19	1.25±0.03	26.32±0.52	1.19±0.04	17.40±0.38	4.32±0.05 (bc)	1.43±0.06 (ab)	13.78±0.45 (b)	5.55±0.33 (d)	3.63±0.12 (bc)	0.39±0.12 (a)
**6th**	21	0.17±0.03	25.53±0.59	1.85±0.03	12.57±0.27	11.73±0.11 (d)	2.68±0.06 (bc)	19.03±0.36 (b)	7.05±0.25 (d)	16.03±0.27 (d)	0.52±0.13 (a)

† Expressed as mean of 3 plants from two experiments;

*values from Figure 3;

**values from Figure 10;

± =  standard error. Different letters in brackets represent statistically significant differences of means (column wise) according to analysis of variance (P<0.05) (Tukey test).

**Figure 5:**
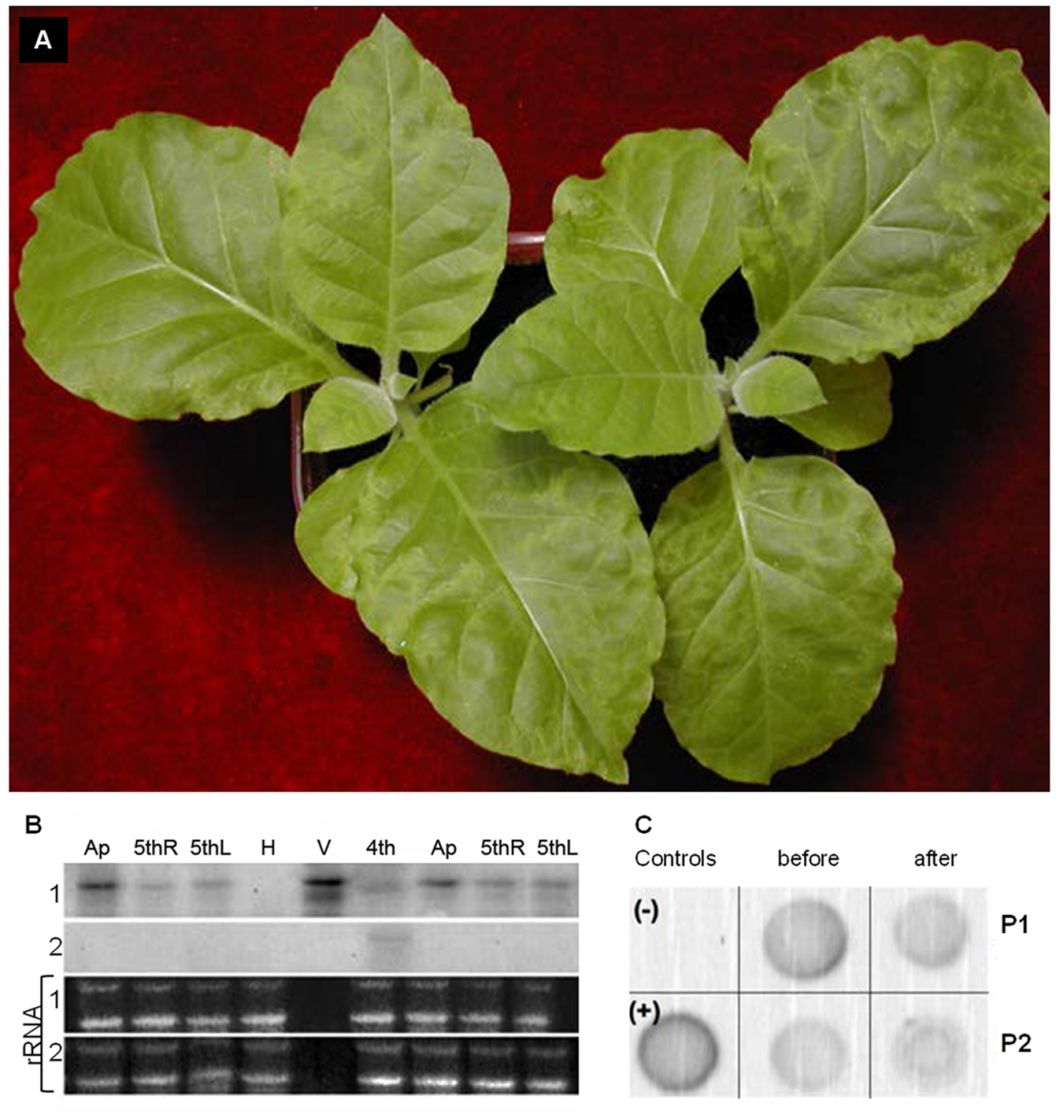
AILV-V would not replicate in leaves recovered from disease symptoms but retains infectivity. In **A.** local and systemic symptoms consisting, respectively, in chlorotic spots and line patterns and, chlorotic/necrotic ringspots surrounding the veins and peripheral vein clearing induced by AILV-V in tobacco at 12 dpi with sap extracted from tobacco leaves, which had recovered from disease symptoms at 40 dpi. In **B.** Northern blot hybridization for detection of (+)RNA (1) and (−)RNA (2) on RNA preparations extracted from the following sources: A =  apical leaves at 40 dpi; 5thR and 5thL =  samples collected from opposite sites (Right and Left) from the 5th leaf of two tobacco plants at 40 dpi with recovery phenotype; 4th =  sample collected from the 4th leaf of a tobacco plant with severe symptoms of systemic infection; V =  RNA from a purified preparation of AILV-V particles, used as positive control; H =  sample collected from an healthy tobacco plant, used as negative control. The picture shows the presence of a weak signal of hybridization with the plus-sense RNA probe (which detects the replication specific minus-sense RNA) only in correspondence of the sample collected from the 4th leaf with severe symptoms of systemic infection. In **C.** Accumulation of AILV-V RNA2 determined by dot blot hybridization in leaf samples collected from two recovered plants (P1 and P2) at 28 dpi before and 14 days after secondary inoculation. + and – indicate positive (pAILV769 plasmid DNA) and negative (mock-inoculated plant leaf) controls, respectively.

AlLV-V was back-inoculated from the leaves recovered from disease symptoms in three AILV-V-infected plants (40 dpi). Sap from each leaf was inoculated onto two tobacco plants. The inoculated plants both developed severe symptoms of local infection by 7 dpi and systemic infection and disease symptoms similar to the plants tested in the experiments described above (Fig 5A). Total RNA extracted from the recovered leaves was tested by northern blot hybridization using a DIG-labeled positive-sense and negative-sense RNA probes to detect the replication-specific, negative-sense RNA and the genomic positive-sense RNA of the viral RNA-2, respectively. The positive RNA-2 strand was detected (Figure 5B, plot 1), but not the negative strand (Figure 5B, plot 2). In contrast, both the negative and positive strands of viral RNA were detected in the 4th leaf showing symptoms.

Recovered leaves appeared resistant to reinoculation with AILV-V, because at 14 dpi there was no increase in AlLV-V RNA-2 amounts in the leaves above the titers detected at the time of inoculation (Figure 5C).

### AILV-V Enters and Persists in the Shoot Apical Meristem of Tobacco Plants

**Figure 6:**
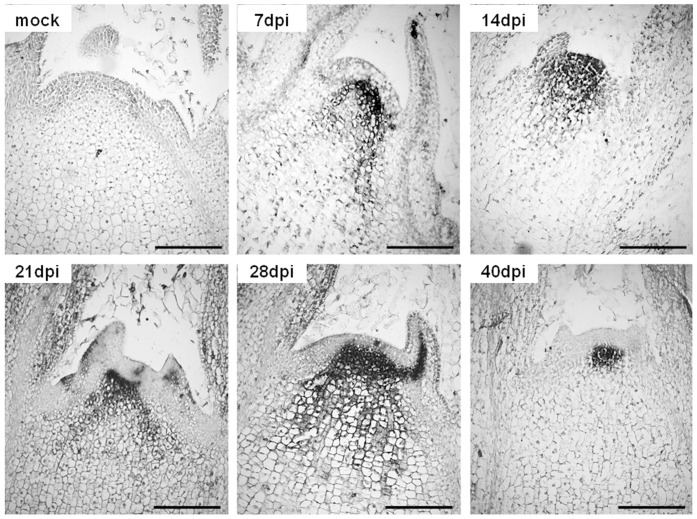
AILV-V enters SAM of Samsun tobacco at a very early stage of infection and persists there up to 40 dpi. One of the two time-course analyses of AILV-V distribution in the SAM of Samsun tobacco showing longitudinal sections of meristem tissues at 7, 14, 21, 28 and 40 dpi. AILV was detected using polyclonal antibodies raised against AILV-V coat protein and signals were developed with alkaline phosphatase diluted 1∶500 in PBS/BSA buffer and stained in NBT/BCIP solution. Immunolocalization is demonstrated in cells with dark stain. Pictures show that between 7 and 28 dpi the virus was present in all meristem tissues and in leaf primordia while by 40 dpi, i.e. concomitantly with the recovery phase from disease symptoms, the virus appeared confined between the corpus and the rib meristem. Mock  =  SAM of healthy tobacco treated with AILV-V antiserum at 21 days after mock- inoculation and used as negative control. Bars = 100 µm.

AILV-V distribution in shoot apical meristem (SAM) of tobacco plants was determined in two independent experiments by immunolocalization in samples collected from eight plants at 7, 14, 21, 28 and 40 dpi and, as control, from eight mock-inoculated plants. After 7 dpi, the virus was detected in the corpus of tobacco SAM, just beneath the tunica layers (Figure 6), and in leaf primordia. Between 7 and 14 dpi, i.e., concomitantly with maximum severity of disease symptoms, the virus invaded also tunica and persisted in the tissues up to 28 dpi, when full invasion of leaf primordia was observed. By 40 dpi, i.e., when the 5th and 6th leaves recovered from disease symptoms, the virus was detected only in few cells just beneath the corpus.

### Transcription profiles of RDR6 and DCL4 during the course of AILV-V infection

To understand how the infected tobacco plants responded to AILV-V infection we analyzed the expression of *RDR*6 and *DCL*4 genes, which are two of the hallmark enzymes of the RS pathway in the study of plant-virus interactions [25], [26]. Variations in the transcription profiles of *RDR6* and *DCL4* (orthologs of *Arabidopsis thaliana*) were monitored in AlLV-V-infected tobacco plants from 10 to 60 dpi in two separate experiments (Figure 3 and Table 1) showing that there was a significant correlation between the amounts of *RDR6* mRNA and accumulation of AILV-V RNA-2 (Figure 3 and Table 1). Until 16 dpi, the expression of *RDR6* did not differ significantly in AlLV-V-infected and mock-inoculated plants but increased rapidly in the 4th leaf between 16 and 18 dpi and reached the maximum in the 5th leaf and 6th leaf at 18 and 21 dpi, respectively. The upregulation of *RDR6* was almost concomitant with the maximal accumulation of viral RNA in the 3rd and 4th leaf (Figure 3 and Table 1) while in the leaves above the 3rd and 4th leaf, *RDR6* expression decreased progressively towards the top of the plant. It was lowest in the 5th and 6th leaf at 23 dpi and reached a steady-state level equivalent to that of mock-inoculated plants between 28 and 60 dpi.

The transcription levels of *DCL4* showed also a progressive increase, which correlated with accumulation of viral RNA, and reached the maximum at 18 dpi in the 5th leaf (Figure 3 and Table 1). During the recovery phase, i.e., when the viral replication diminished progressively until the time point at which it was almost non-detectable, the transcription level of *DCL4* was similar to that of mock-inoculated plants. These results suggested the upregulation of *RDR6* and *DCL4* was a consequence of AILV-V replication and accumulation of its RNA between 10 and 14 dpi in the 3rd and 4th infected leaves.

**Figure 7:**
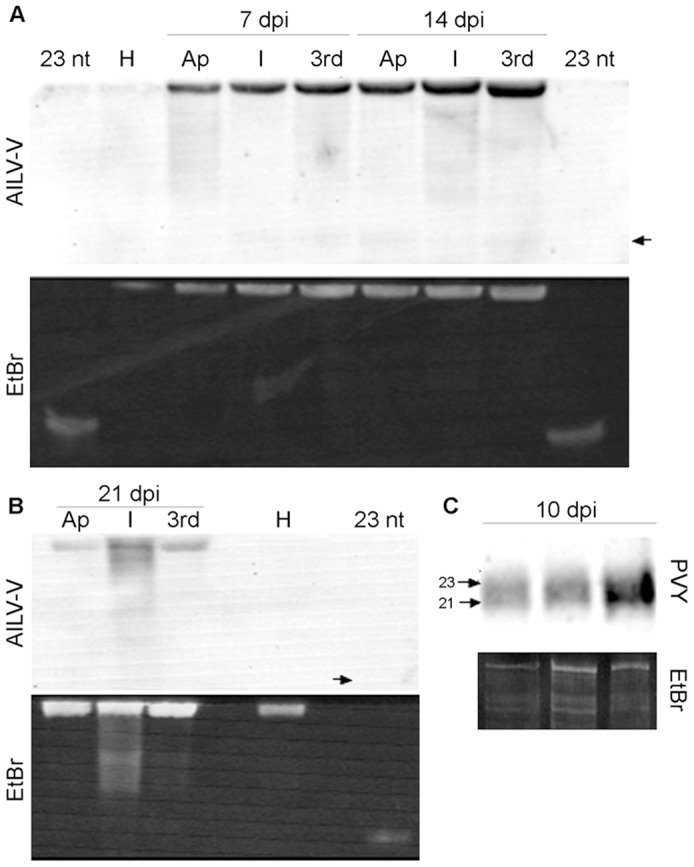
Small interfering RNAs (siRNA) produced in response to AILV-V infection remain below a detectable level. In **A** and **B**, panels represent total RNA preparations enriched in siRNA obtained from samples collected from apical (Ap), rub-inoculated (I) and 3rd (3rd) leaf at 7, 14 and 21 dpi with AILV-V. RNA preparations were first separated in by polyacrylamide gel electrophoresis and stained with ethidium bromide (EtBr)), then transferred to nylon membrane by electroblotting and hybridized with an AILV-V -specific RNA probe (AILV-V) for the last 760 3′-terminal sequences, labeled with digoxigenin and hydrolyzed by alkaline treatment. H =  total RNA extracted from an healthy tobacco leaf. P = 23 bp primer. Arrows point the position expected for the 23 bp primer, after hybridization. In **C**, detection of siRNAs in samples collected from leaves of a tobacco plant at 10 dpi with PVY-SON41. Panels show ethidium bromide staining (EtBr) after PAGE analysis and signals produced after hybridization with a PVY-specific RNA probe (PVY) labeled with digoxigenin and hydrolyzed by alkaline treatment. Arrows point position of 21 and 23 bp primers used as size markers.

The inoculated and systemically infected leaves of tobacco plants were tested for AILV-V specific siRNAs, including plants infected with PVY-SON41 as controls. Despite of two independent experiments carried out to test samples taken at six different time points post-inoculation, it was not possible to clearly detect low-molecular-weight RNA specific for AILV-V (only samples from systemically infected leaves at two representative time points at 7 and 14 dpi are shown in Figure 7A). This was not due to the method we used, because the siRNAs specific to PVY-SON41 were readily detected in plants infected by this virus (Figure 7B).

### AILV-V Is Unable to Prevent the Establishment or Maintenance of RNA Silencing in Response to Infection or to Revert Pre-Established Silencing

To determine whether AILV-V could interfere with the establishment or maintenance of RNA silencing or revert already silenced genes we used a Samsum tobacco transgenic line expressing the HC-Pro silencing suppressor and induced downregulation in the expression of the transgene by the infection of PVY-SON41 as shown previously by Savenkov and Valkonen [27]. Twelve transgenic tobacco plants expressing the PVY HC-Pro silencing suppressor (HC plants) and twelve non-transformed (WT plants) tobacco plants were inoculated with AILV-V, PVY-SON41 or co-inoculated with both viruses and monitored at 14 and 40 dpi for the expression of the HC-Pro protein, the progression of disease symptoms and the accumulation of viral RNAs. Three HC and WT plants were mock-inoculated to serve as negative control.

**Figure 8:**
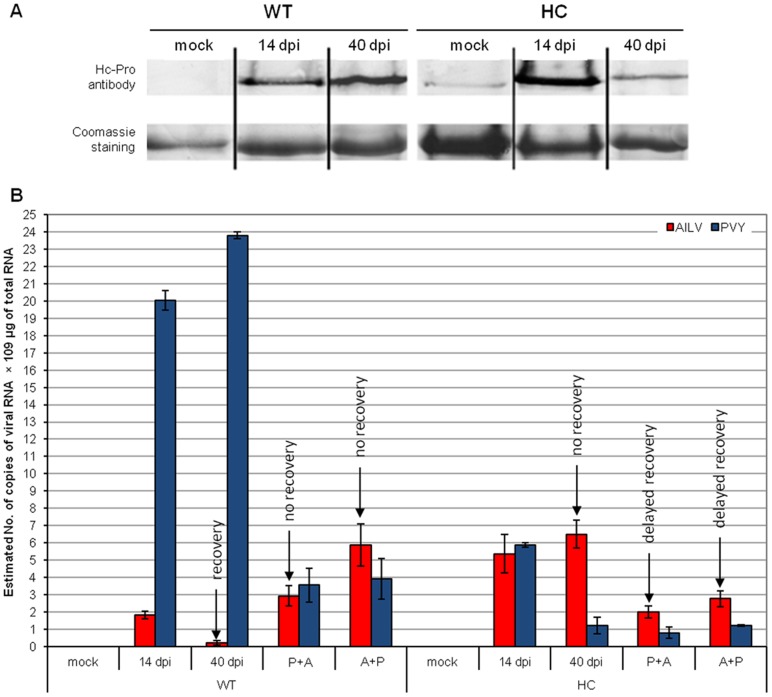
Plants expressing HC-Pro VSR do not enter the recovery phase during AILV-V infection. In **A,** variation in the load of PVY HC-Pro protein detected by western blot in non-transgenic tobacco plants and tobacco plants transformed to express HC-Pro, upon infection with PVY-SON41. WT plants show increasing levels of HC-Pro protein from 14 to 40 dpi while HC-transgenic plants express: i) a steady-state level of the protein after mock-inoculation; ii) an increasing protein load at 14 dpi resulting from the sum of HC-Pro translated from the transgene and from viral transcript and iii) a strong downregulation of the accumulation of the HC-pro protein at 40 dpi caused by the activation of homology-dependent RNA silencing. In **B,** levels of viral RNAs in plants of tobacco wild-type (WT) and transformed with HC-Pro (HC), upon single and mixed infection of AILV-V and PVY-SON41. Columns represent mean values of the number of copies of viral RNAsx10^9^ per µg of total RNA estimated from three biological replicates. Vertical bars represent the standard error. Quantitative dot blot was obtained from the intensity of hybridization signal estimated on the basis of a standard curve generated by serial dilutions of a plasmid preparation containing the RNA fragments targeted by the specific probes. Translation of HC-Pro from either transgenic or authentic virus transcripts favors the infection of AILV-V in WT plants so the plants do not show the recovery phenotype. A+P and P+A indicate the order of inoculation (A = AILV-V and P = PVY-SON41) in mixed infection. Samples were collected 14 days after the second inoculation, corresponding to 40 days from the first inoculation, from the newest fully developed leaves.

In WT plants inoculated with PVY-SON41 the amounts of HC-Pro protein increased from 14 to 40 dpi, whereas in the HC-transgenic plants, a peak of HC-Pro protein accumulation was detected at 14 dpi followed by a drastic reduction by 40 dpi (Figure 8A). The initial increase of HC-Pro protein in HC-transgenic plants was higher than in WT plants and mock-inoculated HC plants and was likely due to the accumulation of HC-Pro translated from the transgene transcript and produced by the infecting virus. The drastic reduction of HC-Pro protein exhibited by HC-transgenic plants at 40 dpi indicated that RS targeted both the HC-homologous transgene and viral sequences.

In this experiment, we examined also the progression of disease symptoms and estimated the loads of viral RNA in single and mixed infections in plants inoculated first with PVY-SON41 or AILV-V and 14 days later with AILV-V (P+A plants) or PVY-SON41 (A+P plants), respectively. WT plants challenged with AILV-V alone showed severe disease symptoms and high viral RNA accumulation at 14 dpi while by 40 dpi plants displayed a recovery phenotype and approx 6-fold reduction of viral RNA accumulation (Figure 8B). In contrast, the abundance of AILV-V RNA in HC plants was 2 to 6-fold higher than in WT plants at the same time points and none of the transgenic plants exhibited recovery up to 40 dpi (Figure 8B). The disease symptoms in WT plants with mixed virus infection were more severe than in plants infected with AILV-V alone and, unlike HC-transgenic plants, they exhibited a delayed recovery phenotype one week later than observed in the plants that were infected with AILV-V only. In addition, the order of inoculation (A+P or P+A) did not affect the disease progression and phenotypic response.

Accumulation levels of PVY-SON41 RNA were also different in WT and HC plants and in plants with single or double infection. In WT plants infected with PVY-SON41 alone, PVY RNA accumulated at very high levels, whereas in plants co-infected with AlLV-V, PVY RNA amounts were 20-fold less and not influenced by the order of inoculation of the two viruses. Accumulation of PVY-SON41 RNA in the HC-transgenic plants was lower in single and mixed infection, regardless of the order of inoculation, than in WT plants infected with PVY-SON41 only (Figure 8B).

Overall these results provide evidence that RS targeted AILV-V, because recovery was delayed in plants expressing PVY HC-Pro while viral RNA concentration was enhanced. In contrast, the virus did not prevent or revert the down regulation of the HC-Pro protein in HC plants upon infection of PVY-SON41 and did not increase accumulation of the potyviral RNA, suggesting poor or weak ability to interfere with RS.

### AILV-V and PVY-SON41 Act Synergistically in Symptom Development but Not in the Accumulation of Viral RNA in Tobacco

**Figure 9:**
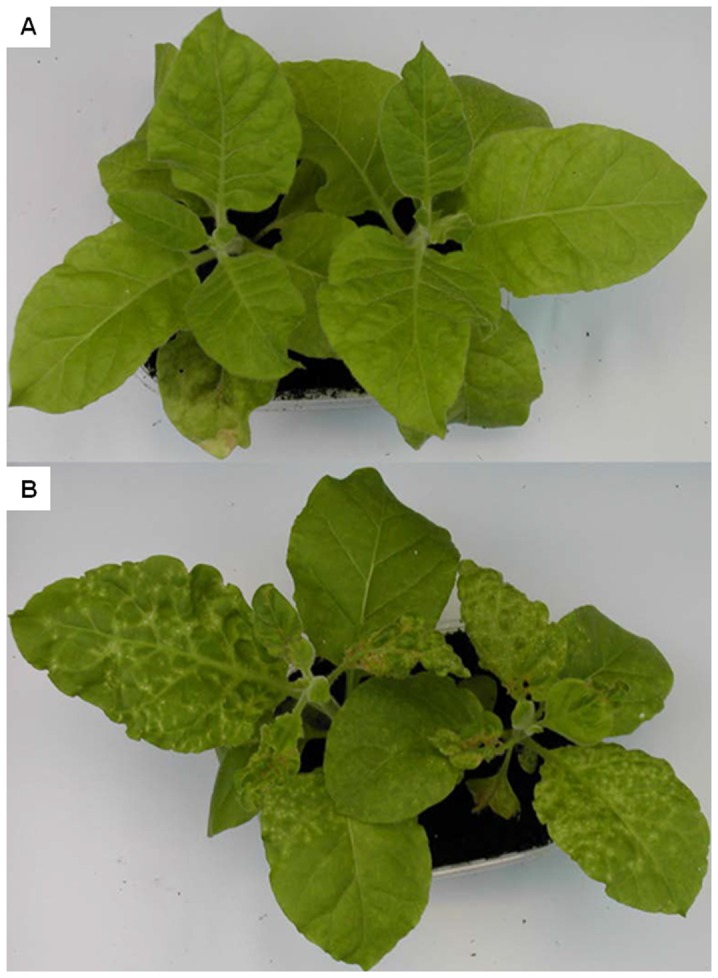
Mixed infections of PVY-SON41 and AILV-V in tobacco exacerbate disease symptoms. In **A,** mild mosaic and moderate leaf blade malformation induced in tobacco at 30 dpi with PVY-SON41. In **B,** Chlorotic/necrotic ringspots, severe reduction of leaf lamina and plant growth induced at 30 dpi by a mixed infection of PVY-SON41 and AILV-V.

**Figure 10:**
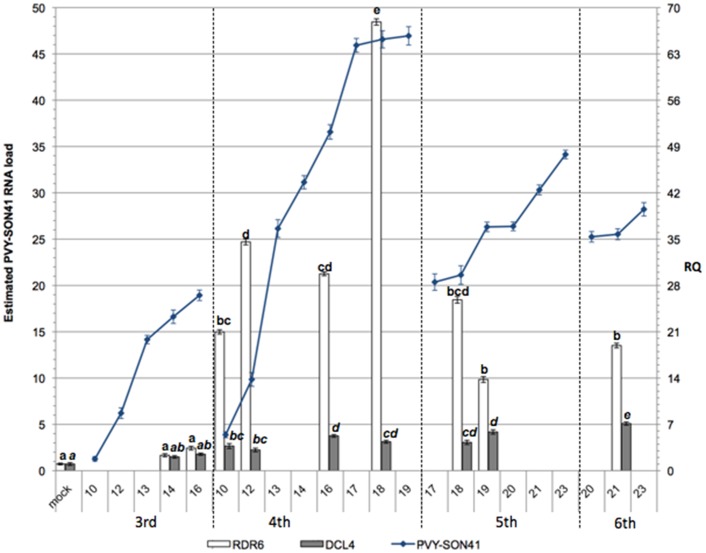
PVY-SON41 infection correlates with viral RNA continuous accumulation and suppression of RNA silencing. Accumulation levels of viral RNA (lines) and transcription profiles of *RDR6* and *DCL4* were estimated by quantitative dot blot hybridization and qPCR, respectively. Symbols and protocols as in Figure 3.

To analyze the trilateral interaction between AILV-V, PVY-SON41 and tobacco in more detail, we monitored symptom development, load of viral RNAs and transcription profile of *RDR6* and *DCL4* between 7 and 21 dpi with AILV-V and PVY-SON41 in single and in mixed infection. AILV-V and PVY-SON41 were inoculated alone, each to a group of 18 plants, while in another group of 18 plants AILV-V and PVY-SON41 were inoculated, respectively, to the 1st and 2nd leaf of tobacco to obtain a mixed infection. Plants with single PVY-SON41 infection manifested symptoms of systemic mosaic, which were persistent in the leaves no. 3 to 6 (Figure 9A and Figure 1). Despite of mild disease symptoms, accumulation of potyviral RNA was high, increased progressively and reached the maximum in the 4th leaf at 17 dpi (Figure 10 and Table 1). After this peak, load of PVY-SON41 RNA decreased and reached a steady-state level in the 6th leaf, which was maintained up to 23 dpi when the monitoring was terminated. The transcription level of *RDR6* followed the pattern of PVY-SON41 RNA accumulation and showed the highest upregulation in the 4th leaf at 18 dpi. Upregulation of *RDR6* in the 5th and 6th leaf was also substantial (Figure 10 and Table 1) but approx 3- to 4-fold lower than in the 4th leaf. In a similar way the expression profile of *DCL4* showed a progressive increase, paralleling the accumulation of PVY-SON41 RNA (Figure 10 and Table 1).

Progression of disease symptoms, accumulation of viral RNAs and transcriptome analysis of *RDR6* and *DCL4* in plants with single AILV-V infection were in good correlation with data obtained from the previous experiment (Table 1). In plants infected with AILV-V at 16 dpi, the transcription level of *RDR6* was 3.5-fold (in the 3rd leaf) to 5-fold (in the 4th leaf) lower than in plants infected by PVY-SON41 (Table 1). This was consistent with the higher RNA loads of the potyvirus. Similarly, the expression of *DCL4* at 16 dpi in plants infected by AILV-V was 2.5-fold lower (in the 4th leaf) than in plants with single infection by PVY-SON41 (Table 1).

Disease symptoms observed at 21 dpi in mixed infected plants were much more severe than those induced by PVY-SON41 and AILV-V in single infections (Figure 9B and Figure 2). However, in the co-infected plants the maximum load of viral PVY-SON41 RNA was reached not earlier than 18 dpi (in the 5th leaf) and was approx 50% of that at the same time point in plants singly infected by the potyvirus, suggesting an inhibitory effect of AILV-V against PVY-SON41 in co-infected tobacco plants. On the other hand, the load of AILV-V RNA in each leaf did not follow the distribution observed in singly infected plants since it increased progressively from the lower to the upper leaves and the maximum load of RNA was reached not earlier than 21 dpi (in the 6th leaf) in the plants infected with AILV-V only (Table 1). Thus, results suggested that AILV-V and PVY-SON41 interfered with each other at the early stages of concomitant infection.

### AILV-V Might Interfere with Cell-to-Cell Movement of the Signal of RNA Silencing

**Figure 11:**
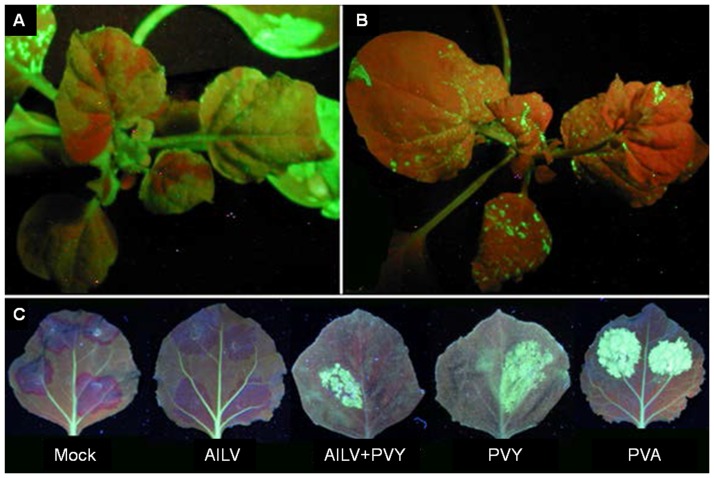
AILV-V is unable to revert GFP silencing while interferes with cell-to-cell movement of silencing signal. In **A,** progression of GFP silencing (indicated by dark red areas along the major veins) in a plant of *N. benthamiana*, line 16c, at 14 dpi with the TMV-GFP vector. Silenced areas were inoculated with AILV-V but no desilencing effects were observed at 30 dpi with AILV-V; rather the silenced areas expanded (in **B**) following the spread of TMV-GFP infection. In **C** Free GFP was expressed transiently in 16c *N. benthamiana* from the binary vector pBIN-mGFP4 carried by *A. tumefaciens*. Prior to agroinfiltration, leaves were mock-inoculated with buffer (Mock) or with AILV-V (AILV), PVY-SON41 (PVY), AILV-V and PVY- SON41 (AILV+PVY) and PVA-B11 (PVA). Upon ectopic expression of GFP, a thin border of dark red tissue was visible at 14 dpi in plants mock-inoculated indicating short-range movement of GFP silencing. This border was not produced in leaves of plants inoculated with AILV-V, suggesting a viral interference with cell-to-cell movement of the silencing signal. Green fluorescent areas visible in AILV+PVY, PVY and PVA infected plants indicate suppression of silencing driven by VSR coded by PVY-SON41 and PVA-B11.

Plant viruses code a wide range of viral silencing suppressor proteins (VSR) targeting different steps of the RNA silencing pathway [26], [28], [29]. To examine the effect of VSR of a number of RNA and DNA viruses on the dynamic of AILV-V infection, plants of *N. tabacum* cv Xanthi transformed with silencing suppressor genes derived from six different viruses [30] were challenged with AILV-V at the five-leaf stage. Twenty plants per each transgenic line were inoculated and symptoms monitored daily up to 50 dpi. Most of the transgenes did not affect recovery from disease symptoms (Table 2). Recovery was prevented only in plants expressing the P1 VSR of *Rice yellow mottle virus* (RYMV, genus *Sobemovirus*) and it was delayed but not prevented in plants expressing the AC2 VSR of *African cassava mosaic virus* (ACMV, genus *Begomovirus*) or HC-Pro of PVY. Because results from experiments with HC-transgenic plants indicated that AILV-V was not able to inhibit establishment and maintenance of RS, we did additional experiments using agroinfiltrated patch assays in the green fluorescent protein (GFP) transgenic plants of *N. benthamiana* line 16c. Systemic silencing in the 16c plants was induced by inoculation of basal mature leaves with infectious transcripts of the engineered clone of *Tobacco mosaic virus* (genus *Tobamovirus*) carrying the GFP gene (TMV-GFP). In non-transgenic plants the expression of *GFP* from TMV resulted in bright fluorescence both in inoculated and top leaves between 4 and 14 dpi while in 16c *N. benthamiana* line, the vector induced silencing of the GFP in most of the leaves, which appeared red under UV illumination due to autofluorescence of chlorophyll (Figure 11A). At 14 dpi with TMV-GFP the red areas were mechanically inoculated with AILV-V and no suppression of silencing was observed up to 40 dpi while the red fluorescent areas continued to expand (Figure 11B).

**Table 2:** Effect of suppressors of RNA silencing (VSR) derived from different RNA and DNA viruses and expressed as transgenes in lines of *N. tabacum* cv Xanthi on the development of symptoms and induction of the recovery phenotype upon infection of AILV-V.

Plant line*	3	7	10	14	17	21	28	31	35	40	50
**WT**	LR	RS	RS	M, R	R	R	R	R	R	R	R
**pBin61**	RS	RS	RS	M, R	R	R	R	R	R	R	R
**P1 CfMV**	LR	RS, VC, M, B	RS, B	RS	RS	R	R	R	R	R	R
**P1 RYMV**	M	RS, VC, M	RS, VC, M	RS, VC, M, B	RS, VC, M, B	RS	RS	RS	RS	RS	RS
**HC-Pro**	LR	RS	RS	RS	RS	RS	RS	R	R	R	R
**AC2**	RS	RS	RS, VC, M	RS, VC, M, B	RS, VC, M, B	RS, B	RS	RS	M	R	R
**P25**	LR	RS, VC, M, B	RS, B	R	R	R	R	R	R	R	R
**2b**	RS	RS, VC, M.5	RS, VC, M, B	RS	R	R	R	R	R	R	R
**P19**	RS	RS, VC, M, B	RS, B	RS	RS	RS	R	R	R	R	R

*Transgenic plant line expressing the following VSR: P1 from *Cocksfoot mottle virus* (P1 CfMV) and from *Rice yellow mottle virus* (P1 RYMV), Hc-Pro from Potato virus Y (PVY), AC2 of *African cassava mosaic* (ACMV), P25 from *Potato virus X* (PVX), 2b from *Cucumber mosaic virus* (CMV) and P19 from *Tomato bushy stunt virus* (TBSV). Symptoms were recorded between 3 and 50 dpi with AILV-V. VC =  vein clearing; LR =  local ringspots; RS = systemic ringspots; M =  mosaic; B =  leaf blistering R =  recovery. WT =  untransformed *N. tabacum* cv Xanthi; pBin61 =  empty vector.

In patch assays, the 16c plants were rub-inoculated either with AILV-V, PVY-SON41 or strain B11 of *Potato virus A* (PVA-B11, genus *Potyvirus*) alone, or co-inoculated with AILV-V and PVY-SON41. At 7 dpi, fully enlarged leaves of *N. benthamiana* were agroinfiltrated on the opposite sides of the midrib with *A. tumefaciens* containing the binary vector pBIN-mGFP4 for the expression of GFP under *Cauliflower mosaic virus* 35S promoter (35S). Upon ectopic expression of GFP, a thin border of dark red tissue was visible at 14 dpi in mock-inoculated plants, indicating short-range movement of GFP silencing (Figure 11C). However, the border was not observed in leaves of plants inoculated with AILV-V, suggesting that the virus interfered with cell-to-cell movement of the silencing signal. Green fluorescent areas without red borders visible in leaves inoculated with AILV+PVY, PVY or PVA indicated suppression of silencing driven by VSR coded by PVY-SON41 and PVA-B11 (Figure 11C).

## Discussion

This study investigated the dynamics of AILV-V infection in tobacco revealing the following new insights in nepoviral life-cycle: the virus colonized the SAM at 7 dpi and persisted in meristem tissues up to 40 dpi while new leaves showing recovery from severe viral symptoms did not appear before 21 dpi. The asymptomatic leaves, which developed during the recovery phase, contained infectious virus particles but no replication of AILV-V was apparent, and the leaves were protected against reinoculation with AILV-V. We therefore hypothesize that the recovery from symptoms observed in the top leaves might result from a defense response, which, like with PVX [14], was not primed by the entry of AILV in the SAM but was triggered in tissues of systemically infected leaves as soon as accumulation of viral RNA reached a threshold level. The progression of AILV-V infection in tobacco plants was similar to that of the W22 strain of *Tomato black ring virus* (TBRV-W22) [15], another nepovirus of the subgroup B, but it differed from TRSV and ToRSV, which are nepoviruses belonging to subgroup A and C, respectively [6]. Therefore it seems that the dynamics of nepovirus entry and persistence in tobacco SAM, as well as viral RNA accumulation and persistence in asymptomatic leaves recovered from disease symptom, are different among virus species belonging to distinct subgroups of the genus *Nepovirus*.

Quantitative dot blot and immunolocalization analyses provided evidence that AILV-V entered SAM of tobacco plants within 7 dpi and persisted detectable therein during the recovery phase at least up to 40 dpi. Time-course experiments shown in Figure 4 demonstrated that from inoculated leaves the virus moved fastest to the topmost leaf (apical leaf), where it was found at 5 dpi, whereas it was detected in the 3rd leaf (i.e., the first leaf above the two inoculated leaves) not before 7 dpi when the virus was immunolocalized also in the SAM. According to the model proposed by Foster *et al*. [13] and Schwach *et al*. [14] invasion of meristematic tissues should have been prevented by the *RDR*6-mediated amplification of the systemic silencing signal, which was not the case with AILV-V as the virus was detected in SAM one week before *RDR6* was overexpressed. Therefore, we hypothesize that similarly to TRSV [10], entry of AILV-V in the meristematic tissues was permitted by the very low concentration of viral RNA or virus particles at a time of infection that would be insufficient to trigger an RS-mediated response. Results of immunolocalization between 7 and 28 dpi showed a higher concentration of virus in meristem than in surrounding tissues, as with TRV and TRSV [16], [19], suggesting poor or no AILV-V replication and movement in the SAM. The successive reduction of virus titer observed in SAM by 40 dpi might be due to hindered viral transport into the meristem as a consequence of virus clearance in tissues of mature leaves. This model is supported also by the fact that we were unable to demonstrate viral replication in leaves fully recovered from disease symptoms, although these tissues contained viral particles that proved highly infectious in back-inoculation experiments. However, as found in previous studies [31], [32], [33] we cannot exclude that AILV-V may have replicated in groups of cells.

Roberts et al. [34] proposed that recovery from ringspot symptoms induced by TRSV occurred after viral invasion of the SAM and Ratcliff et al. [15] linked the appearance of the recovery phenotype from TBRV infection in *N. benthamiana* to the activation of RS, which, in turn, was correlated with viral invasion of the SAM. Conversely, the model proposed for TRV in *N. benthamiana* implies that fully expanded leaves showing the recovery phenotype are those deriving from SAM after virus clearance [16]. With AILV-V we provided evidence that the virus was present in tobacco SAM at least two weeks before initiation of the recovery phase in leaves and that reduction of virus titer observed in SAM at 40 dpi was concomitant with the steady-state level of viral RNA loads during the recovery phase in mature leaves. Therefore, we propose that with AILV-V, invasion of the SAM and initiation of recovery are spaced-out processes. Collectively, the model of SAM invasion proposed for AILV-V is consistent with the pattern of meristem invasion in *N. benthamiana* plants proposed for TRV [16]. However, while TRV infection was supported only transiently in meristematic tissues and by the activity of its VSR, with AILV-V we propose that the virus persisted in meristem tissues because of the very low level of accumulation, which was not sufficient to trigger RS. Nonetheless, the activity of a hitherto unknown VSR coded by AILV-V, accounting for its persistence in meristematic tissues, cannot be excluded.

Results of time-course experiments (Figure 3 and Table 1) showed variations in the expression profiles of *RDR6* and *DCL4* during AILV-V infection and suggested activation of an RS-based host response after a threshold of viral RNA concentration was reached between 10 and 14 dpi which coincided with systemic infection of the 3rd leaf (first leaf above the inoculated leaves). We were unable to detect AILV-V- specific siRNAs as evidence for antiviral RS, as reported with plants infected by ToRSV [20] and TRSV [10] as well as in other plant-virus combinations [35], [36]. However, indirect evidence that AILV-V was targeted by RS was obtained by the analysis of transgenic lines expressing suppressors of silencing and from the plants in which AILV-V was in mixed infection with PVY. Recovery was delayed in plants expressing PVY HC-Pro and AILV-V RNA concentration was enhanced and was prevented as well in transgenic plants expressing AC2 or RYMV P1. We therefore hypothesize that, similar to TBRV and TRSV [10], [15], induction of the recovery phenotype in plants infected by AILV-V might be a consequence of RS activated in lower leaves, which conditioned negatively the accumulation of viral RNA in all the leaves that developed later.

The transcript profile of *DCL4* increased consistently with the accumulation of AILV-V RNA, although with smaller variations than *RDR6*. In the plant response to viral infections *DCL4* is involved in biogenesis of the bulk of viral siRNAs by dicing perfectly paired double-stranded RNAs generated by either the viral replicase or host-encoded *RDR*6 and *RDR*1 [26], [37]. Upregulation of the transcription levels of *DCL4* was therefore expected at the time points in which maximum accumulation of viral RNA and maximum expression of *RDR6* were recorded, but only modest if any changes were observed. This might be taken as indirect evidence that AILV-V interferes with the activity of *DCL*4 in the RS process.

Finally, although the agroinfiltrated patch assay suggested that AILV-V would be able to interfere with cell-to-cell movement of the silencing signal, further experimentation will be required to determine if the AILV-V genome encodes a suppressor of RS and define its mode of action.

## Materials and Methods

### Plants and Viruses

Plants of *Nicotiana tabacum* cv. Samsun (tobacco) were used in the experiments. Transgenic plants of tobacco cv. Samsun expressing the helper component proteinase (HC) of *Potato virus Y* (PVY) were obtained from Dr. Peter Palukaitis (Department of Horticultural Sciences, Seoul Women's University, Seoul, South Korea) while transgenic *N. benthamiana* line 16c [38] was kindly provided by Dr. David Baulcombe (Department of Plant Sciences, University of Cambridge, Cambridge, United Kingdom). Lines of tobacco cv. Xanthi transformed with genes for the expression of the following viral RNA silencing suppressor (VRSs) [29] were also used: P1 of *Rice yellow mottle virus* (RYMV), P1 of *Cocksfoot mottle virus* (CoMV), P19 of *Tomato bushy stunt virus*, (TBSV), P25 of *Potato virus X*, (PVX), HC-Pro of PVY, strain N (PVY), 2b of *Cucumber mosaic virus*, strain Kin (CMV), AC2 of *African cassava mosaic virus* (ACMV). Plants were grown in a glasshouse at 24±2°C with a 16 h light and 8 h dark regime. Inocula were prepared by crushing systemically infected leaves in 100 mM Na_2_-K phosphate buffer, pH 7.2, containing 1 mM sodium sulphite and by rubbing the extracts onto celite-dusted 1st and 2nd completely unfolded true leaves (approx. growth stage 1002 of the scale for coding growth stages in tobacco – coresta at http://www.docstoc.com/docs/155125965/A-Scale-for-Coding-Growth-Stages-in-Tobacco---coresta and Figure 1). Plants were screened daily for symptoms appearance and sampled at 7, 14, 21, 28, 40 and 60 days post-inoculation (dpi) for molecular assays. The following inocula were used: the grapevine isolate of AILV (AILV-V) [39]; the SON41 isolate of PVY (PVY-SON41) kindly provided by B. Moury (INRA, Montfavet, France); an isolate of *Potato virus A* strain B11 (PVA-B11) [40]; and a biologically active transcripts of a recombinant *Tobacco mosaic virus* vector, denoted TMV-GFP, carrying the ORF of the green fluorescent protein (GFP) from the jellyfish (*Aquorea victoria*). Inocula of AILV-V and PVY-SON41 were obtained from systemically infected Samsun tobacco plants while inoculum of PVA-B11 was maintained in Xanthi tobacco plants. Infectious transcripts of TMV-GFP were synthesized from the plasmid pBSG1057 linearized at its *Kpn*I site, using the T7 RNA polymerase and the mMessage mMachine kit (Ambion, Austin, TX, USA), following the protocol of the manufacturer. The plasmid pBSG1057-5 (kindly supplied by Dr. Helene Belanger, Large Scale Biology Corporation, Vacaville CA, USA) contains the TMV genome including the GFP ORF placed under the control of a duplicate of the TMV coat protein subgenomic promoter. Ten microliters of the transcription mixture were rub-inoculated onto each leaf of *N. benthamiana* 16c plants. Plants mock-inoculated with buffer served as negative control.

### RNA Extraction and Analysis

Total RNA was extracted from 50 mg plant tissues with the TRIzol reagent (Invitrogen, San Diego, CA, USA) as described by the manufacturer and subjected to RQ1 DNase digestion (Promega, Madison, WI, USA). RNA preparations were suspended in 30 µl RNase-free water and concentration and quality estimated with a Nanodrop ND-1000 spectrophotometer (Nanodrop Technologies, Rockland, DE, USA), electrophoresis through 1.2% agarose gel in TBE buffer (90 mM Tris, 90 mM boric acid, 1 mM EDTA) and Gel-red (Biotium, USA) staining. Final RNA concentrations were adjusted to 1 µg/µl. Plant response to the single and mixed infection of AILV-V and PVY-SON41 was evaluated by the quantification of the expression level of the genes for *RDR*6 and *DCL*4 in time-course experiments with reverse transcription quantitative real-time PCR (qPCR), using glyceraldehyde 3-phosphate dehydrogenase (*GAPDH*) as housekeeping gene [41]. A group of four plants was inoculated with AILV-V, another group was inoculated with PVY-SON41 and a third group was inoculated with both AILV-V and PVY-SON41 on the1st and 2nd completely unfolded true leaves of the same plant. Total RNA was extracted from 50 mg leaf tissue collected daily from the 3rd to the 6th leaf between 10 and 60 dpi. In particular, samples from the 3rd and the 4th leaf were collected between 10 and 16 dpi; those from the 4th and 5th leaf between 17 and 19 dpi and those from the 5th and the 6th leaf between 20 and 23 dpi. The 6th leaf was used also to collect samples at 28 and 60 dpi. Total RNA extracted from mock-inoculated plants at each sampling time was used as control. Primers pairs used for the amplification of *RDR*6, *DCL*4 and *GAPDH* transcripts were designed with Primer-BLAST (http://www.ncbi.nlm.nih.gov/tools/primer-blast/) and were *RDR*6 For (5′-CGACCTCGCAATGGTCCTA GC-3′) and *RDR*6 Rev (5′-GCTCGTCCTTCACCCGGAGC-3′), designed on the basis of GenBank Accession No AB361628; *DCL*4 For (5′-GTTGACAAGTGGCTTCAAAGCAG-3′) and *DCL*4 Rev (5′- CTTTGCTTGCAGCGCAAATACTC-3′), designed on a Blast alignment between sequence FM986783 from *N. benthamiana* and sequence AM846087 from *N. tabacum* and *GAPDH* For (5′-CGGCCGCCCCGTTGTATC-3′) and *GAPDH* Rev (5′-GAGAGGAGGAGCGAAGTCC-3′), designed on the basis of GenBank accession no. AJ133422). First strand cDNAs were synthesized from 1 µg of total RNA preparation and 10 pmol random hexamers with High Capacity cDNA Reverse Transcription kit (Applied Biosystems, Foster City, CA, USA) following the protocol of the manufacturer. qPCR was set up in 10 µl of 2X Fast SYBR Green PCR Master Mix (Applied Biosystems), containing 100 ng of first strand cDNA template, and 200 nM each of the forward and reverse primer pairs. Each cDNA sample was amplified in triplicate on a single 48-well optical plate using the StepOne Real-Time PCR system (Applied Biosystems). The cycling profile consisted of 95°C for 2 min followed by 40 cycles of 3 s at 95°C and 10 s at 60°C. Immediately after the final PCR cycle, a melting curve analysis was done to determine the specificity of the reaction. Relative quantification was calculated using the comparative cycle threshold (Ct) method (RQ = 2^−ΔΔCt^) [42], in which the change in the amount of the target viral RNA was normalized in relation to the endogenous control. Validation experiments were done according to the manufacturer's instructions (Applied Biosystems) to compare the amplification efficiencies of *RDR*6 and *DCL*4 and the endogenous *GAPDH* mRNA primers. The experiment was repeated twice and statistical significance of the RQ values was assessed by one-way analysis of variance (ANOVA) with Tukey post-hoc test (P<0.05), for each target gene separately, to compare gene expression at each time point.

### SiRNA Detection

Preparations of total RNA extracted from leaves of tobacco plants collected daily from 1 to 7 and then at 14 and 21 dpi with either AILV-V or PVY-SON41 were enriched in low-molecular-weight plant RNAs by differential precipitation as described by Bucher et al. [43] with minor modifications. RNAs were separated by denaturing 15% polyacrylamide gel electrophoresis, transferred to positively charged Nylon membranes (Roche) by electroblotting as described by Cillo et al. [44] and cross-linked to the membrane under UV light for 3 min. Low-molecular-weight plant RNAs prepared from tobacco plants infected with PVY-SON41 were used as control. Membranes were hybridized with digoxigenin-labeled RNA probes specific for the last 760 bp and 1800 bp 3′-terminal sequences of AILV-V and PVY-SON41, respectively, and hydrolyzed to small fragments at 60°C for 50 min in 200 mM NaHCO_3_ and 200 mM Na_2_CO_3_. After incubation, the solution was neutralized with acetic acid. Hybridizations and washes were conducted at 42°C. Chemiluminescent signal yielded by hybrids was acquired with 5 min intervals for 90 min of exposure in a ChemiDoc (Bio-Rad Laboratories).

### Dot Blot Hybridization Analysis

Dot-blot hybridization was used to verify the presence and estimate the loads of viral RNA in plant tissues. Samples were homogenized with six volumes (v/w) of alkaline solution (50 mM NaOH, 2.5 mM EDTA) and 5 µl from the homogenate were directly applied onto a positively charged Nylon membrane (Roche Diagnostics, Mannheim, Germany) and fixed by UV exposure. Digoxigenin-labeled DNA probes for AILV-V and PVY-SON41 were prepared from plasmids pAILV769 and pPVY-SON41-617, respectively, and used as described previously [45]. Plasmid DNA and unincorporated nucleotides were removed, respectively, using the Whatman FTA kit pK1 (Whatman) and Bio-Rad Bio spin P30 columns (Bio-Rad Laboratories) following the manufacturer's protocol while PCR products were anlayzed for purity by agarose gel electrophoresis. For quantitative dot blot analysis, three biological replicates of samples collected at each sampling time were used and each sample was spotted as two technical replicates. Reproducibility of hybridization signals among biological replicates and samples collected at different time points was assessed by a preliminary dot blot hybridization using *GAPDH* as target and a specific Digoxigenin-labeled DNA probe. A standard curve was obtained by the intensity values of the hybridization signals produced by dilution series of unlabeled PCR products derived from the same insert used to synthesize the probe. Concentration of unlabeled PCR products was estimated with a Nanodrop ND-1000 spectrophotometer. Chemiluminescent signals were acquired after 15 minutes of exposure in a ChemiDoc (Bio-Rad Laboratories), and their intensity estimated by the Quantity One software (Bio-Rad Laboratories, Berkeley, CA, USA).

### Immunolocalization

Shoot tips were collected from tobacco plants at weekly intervals, fixed overnight at 4°C with 4% paraformaldehyde in PBS (137 mM NaCl, 2.7 mM, 10 mM Na_2_HPO4, 2 mM KH_2_PO4, pH 7.4) and dehydrated in an incremental (30, 50, 70, 85 and 99%) ethanol series. Samples were cleared in Histo-clear (Natural Diagnostics, Atlanta, GA, USA) and embedded in paraffin (Paraplast; Sigma-Aldrich, St. Louis, MO, USA). Semithin longitudinal sections (10 µm) were collected on polysine slides (Thermo scientific, Braunschweig, Germany) and incubated for 16–18 hours at 37°C. For immunolocalization, sections were treated with Histo-clear for 3 min to remove paraffin, hydrated in ethanol series, pre-incubated with PBS containing 4% bovine serum albumin (BSA) for 30 min and incubated for 3 h at room temperature with a polyclonal serum raised in rabbit against AILV-V diluted 1∶500 in PBS. After washing in PBS three times, the samples were incubated with the secondary mouse-anti rabbit monoclonal antibody conjugated with alkaline phosphatase (Sigma Aldrich, St. Louis, MO, USA) diluted 1∶500 in PBS/BSA buffer and stained in NBT/BCIP solution (Sigma-Aldrich) following the protocol of the manufacturer. Slides were observed with a light microscope and pictures taken with a camera integrated to the microscope.

### Local Suppression of Silencing in Agroinfiltrated Patch Assays

The *GFP* reporter gene from the pBin-GFPsense pBIN-mGFP4 binary vector [46] was expressed transiently in leaves of *N. benthamiana*, line 16c, by infiltration of a culture of *Agrobacterium tumefaciens* carrying the recombinant plasmid. Prior to agroinfiltration, leaves were either mock-inoculated with buffer or with PVA, AILV-V or PVY-SON41, or co-inoculated with AILV-V and PVY- SON41. To monitor the effect of different viral inocula on the suppression of RNA silencing, leaves were illuminated with a Black Ray long wave UV lamp (UVP, Upland, CA, U.S.A.).
